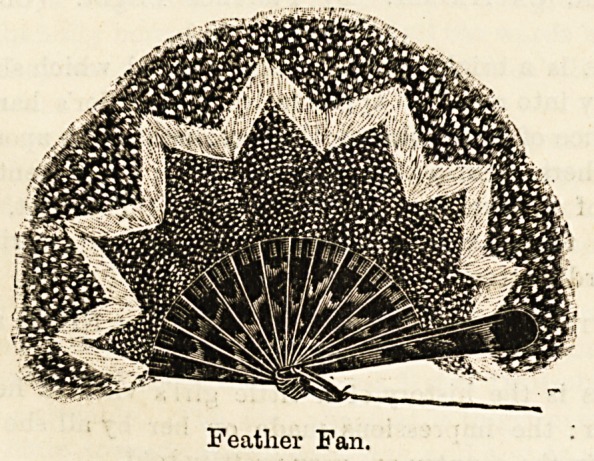# The Hospital. Nursing Section

**Published:** 1902-12-13

**Authors:** 


### The Hospital.

### Nursing Section.

Contributions for this Section of "THE HOSPITAL" should be addressed to the EDITOR, "THE HOSPITAL" NURSING SECTION, 28 & 29 Southampton Street, Strand, London, W.C.

No. 846.—VOL. XXXIII. *SATURDAY, DECEMBER* 13, 1902.

### Notes on News from the Nursing World.

### A REMINISCENCE OF QUEEN VICTORIA.

THE great importance which Queen Victoria attached to good nursing is brought out in a striking manner in Mr. Sidney Lee's Biography of the late Sovereign just issued. Mr. Lee states that when the present king recovered from his illness in 1871, Queen Victoria, "in private talk," attributed the happy issue to the careful nursing of the patient, and sadly remarkd "Had *my* Prince had the same treatment as the Prince of Wales he might not have died." This feeling had probably a good deal to do with her Majesty's decision to devote the sum given her by the women of England to the foundation of the Jubilee Institute, in order that the sick poor might enjoy advantages which were not available to them in 1861.

### THE ROYAL RED CROSS

THE Secretary of State for War wishes us to intimate that ladies now in England who have been awarded the Royal Red Cross, but who may not have received the decoration, should communicate their addresses immediately to the Under Secretary.

### RESERVE SISTERS AND PENSION.

IT is officially announced that sisters of the Army Nursing Service Reserve who have served with an army in the field, or who have been employed on army service at home, shall, if they are selected for Queen Alexandra's Imperial Military Nursing Service, be permitted to count such service towards pension. This is only fair, but it is rather curious that the notice was not issued by the War Office until now.

### OUR CHRISTMAS DISTRIBUTION.

THE articles received by us for distribution at Christmas to the patients in hospitals and infirmaries will be on view at the offices of THE HOSPITAL, 28 and 29 Southampton Street, Strand, on Tuesday next, December 16. Nurses who desire to see for themselves the contributions which have been so generously sent to us will be welcome between 3 and 5 P.M. We have to acknowledge, with many thanks, parcels from :—M. R.; "An Old Lady of 92"; Miss Hitchcock, The Beeches, King's Lynn; and "A Grateful One," 2s. 6d. It is possible that there may be still readers who have not yet forwarded intended contributions. If so, their parcels should reach the Editor of THE HOSPITAL not later than Monday next, December 15.

### HALF-TRAINED NURSES AT HARTLEPOOLS HOSPITAL.

AT the last monthly meeting of the governors of Hartlepools Hospital, the chairman, Dr. Morison, in urging the appointment of another nurse, said that they had been promoting nurses with two years' training to the responsible position of ward nurse, and they had been in their turn responsible for the training of the probationers. "Thus," he added, "they had been putting half-trained nurses to train those who had no training at all." He urged that the best thing would be not to promote any nurse until she had served three years' probationership, and we are glad to add that a resolution which will end the practice condemned by the chairman, was carried by a handsome majority, in spite of a protest on the part of its supporters.

### THE JUBILEE INSTITUTE AND A PROVIDENT BASIS.

IN connection with a movement at Bishop Auckland to remodel the rules of the District Nursing Association in the town with the view of reforming it on a permanent basis, we may remark that branches of Queen Victoria's Jubilee Institute for Nurses are not at liberty to radically alter their regulations as they please. Of course, they can cut themselves off from the parent organisation if they choose, but the proposal included in the scheme of reform at Bishop Auckland forbidding a nurse to attend any case except the patient, or the head of the family, is a member paying 2s. a year, or a halfpenny a week, is out of harmony with the principle on which the Jubilee Institute was founded. However desirable it is that the working classes should be induced to become provident, to refuse to nurse the sick because they have been improvident is not a policy that the Council of the Institute could sanction.

### DEVON AND EXETER HOSPITAL.

THE President of the Royal Devon and Exeter Hospital has received an intimation from Dr. Cuthbert J. S. Wallace, of 26 Upper Wimpole Street, to the effect that having examined the probationers of the institution by means of a written paper and *vivâ voce* examination, he is able to report that "the standard reached was an exceedingly good one throughout," and that "the best candidates attained a very high level." Dr. Wallace adds that all the nurses showed signs of thorough and careful teaching as well as a good knowledge of modern methods in both medical and surgical nursing.

### THE IRISH FIASCO.

THE Executive Committee of the "Coronation National Fund for Nurses in Ireland" have published a letter from Sir Algernon Coote generously offering to give another £100 to the fund if £900 more can be raised "in any way" by December 31st, or £145

more if the total fund can be raised to £5,000 by the same date. Sir Algernon adds, "It is the very greatest pity that this excellent fund should not be a success." But even if the £5,000 were raised by the end of the month it would, we fear, remain a tremendous failure as a provision for the 3,860 nurses in the sister island, who, judging from a correspondent's statement in our last week's issue, are not in a position to pay the premium of the Royal National Pension Fund for Nurses. According to the same correspondent, there is now talk of introducing a rule to grant aid to members to take out policies in the National Pension Fund. If something of this sort had been proposed at the outset, a more cordial reception might have been given to the movement.

### DISTRICT NURSES AND OUTDOOR RELIEF CASES.

At the quarterly meeting of the Grimsby and District Nurses' Institution the secretary was requested to write to the Grimsby Board of Guardians to ask for an increased grant. It appears that the time of one nurse is almost entirely occupied in attending to patients receiving outdoor relief, and as the sum subscribed by the guardians is only £15 15s. per annum, the committee of the Institution are justified in requiring an increase as the condition of continuing to send a nurse to the cases. The demand is all the more reasonable because the nurses employed by the Institution are all fully trained. For such circumstances as obtain at Grimsby, guardians can well afford to pay the substance of the salary of the district nurse.

### A DOCTOR BOYCOTTS A NURSE.

A very unusual situation has arisen at Naas in connection with the District Nursing Association. The dispensary medical officer, Dr. Murphy, who was only appointed a little time ago, refuses to avail himself of the services of the district nurse for his patients. He is obviously within his rights in pursuing this course. But his action was severely criticised at a special meeting of the Nursing Association. The nurse employed by that body, Miss A. A. Short, has occupied her present position for seven years, to the complete satisfaction of the organisation, among her warmest friends being Dr. Smyth, a leading medical man; and a resolution expressing entire confidence in her has been unanimously passed. Dr. Murphy's defence of his conduct is that he has substituted his own attendance on the sick under his charge for that of the nurse, and that he objects to her on personal grounds. As it was explicitly stated at the meeting that in her long period of service not a single fault had been found with Miss Short, the personal objection seems exceedingly ungracious.

### A SUBSTANTIAL BALANCE AT LLANDUDNO

The annual report of the Llandudno Nursing Association, which was adopted at the meeting, shows that the receipts, including the balance carried forward from last year, amounted to £285, and the expenditure to £114. The balance, it will be observed, is very substantial, and indicates that the residents of the popular seaside resort in North Wales not only admire the work of the Queen's nurse, but are determined that lack of funds shall not be a difficulty. During the year which ended September 30th, the nurse was sent to 180 cases by the local doctors, and paid 3,270 visits. As a proof of the general interest taken by the public, it may be added that the Amateur Dramatic Society sent two contributions of £7 16s. 6d. and £2 3s. 6d. to the honorary secretary who also received a cheque for £3 4s. 2d., part of the proceeds of a theatrical entertainment at the Prince's Theatre.

### DISTRICT NURSING AND NIGHT WORK.

The secretary of the Matlock Nursing Association has been compelled to intimate to the people in the neighbourhood that the district nurse must not be called out in the night in the event of death or for any other case except notice be sent direct to her from one of the doctors. It appears that thoughtless people have been in the habit of sending for her in the night to attend the most trivial cases. A district nurse is generally ready to go wherever and whenever her services are needed, but it is obvious that she cannot work day and night. Her hard-earned rest should not be lightly broken.

### A START AT MERE.

It has been decided to form a branch of the Queen Victoria's Jubilee Institute for Nurses in the town of Mere. At a meeting in support of the movement a representative provisional committee was formed, including the vicar and the Congregational minister; and it was announced that the Duchess of Somersetshire had offered to subscribe £30 for the first year, and had promised the proceeds of an amateur dramatic entertainment.

### AN INSTITUTE FOR WELSHPOOL.

Thanks to the generosity of the Misses Howell, of Rhiewport, who volunteered a site for the Victoria Nursing Institute at Welshpool, the foundation stone of the building has been laid. Moreover, before the proceedings at the ceremony closed, Mr. Charles Howell, representing the family, placed a bank note for £500 on the stone in aid of the endowment fund. Other substantial subscriptions were announced, and it seems probable that by the time the Institute is finished, a handsome sum for this purpose will be at the disposal of the Association. At present the nurses live in an inconvenient house, but the new building will be on a suitable scale, and in addition to providing every necessary accommodation for the staff, will contain two wards, each for three beds.

### THE OLD HILL NURSING ASSOCIATION.

The first year's work of the Old Hill Nursing Association which has just been completed has fully justified its existence. The district is almost entirely an industrial one and the need of effective and trained nursing had long been felt. A committee of ladies was formed, and during twelve months they raised the sum of £81 16s. 8d., subscribed to a large extent by small contributions from the working classes. The necessity for a district nurse has been demonstrated by the fact that during the first nine months Miss Leng paid 3,287 visits. Subscriptions already received justify the hope that the association will be still more financially prosperous in the future. Many districts fear to enter upon nursing schemes because of the difficulty of raising the necessary funds, but the experience of the Old Hill Nursing Association may encourage similar localities to follow its example.

### The Midwives Act.

By Honnor Morten.

There are one or two practical points with regard to the Midwives Act, 1902, to which it is worth while to call attention. First, that the only certificates recognised in clause 1 are the London Obstetrical Societies and three Irish ones. Here is the wording of the clause:—

"Any woman who, within two years from the date of this Act coming into operation, claims to be certified under this Act, shall be so certified provided she holds a certificate in midwifery from the Royal College of Physicians of Ireland, or from the Obstetrical Society of London, or the Coombe Lying-in-Hospital and Guinness's Dispensary, or the Rotunda Hospital for the Relief of the Poor Lying-in Women of Dublin, or such other certificate as may be approved by the Central Midwives' Board, or produces evidence, satisfactory to the Board, that at the passing of this Act, she had been for at least one year in *bonâ fide* practice as a midwife and that she bears a good character."

It leaves open the adding of other certificates to the Central Board, and they will surely recognise some Scotch certificate, but the fact remains that it is most important for any midwife training now in England to secure the L O S. certificate and not to be put off by one given by a lying-in hospital.

Secondly, the power left to the Central Board just appointed is very great; it will be the drawing up of all the rules and regulations under which the midwives will work, and these may be vexatious or otherwise according to the pressure brought to bear upon the board. It is well, then, to remember that the three women on the board are Miss Wilson, Miss Paget, and Miss Oldham, and that through them probably any practical points could be impressed on their fellow members. It is midwives who have worked long in country districts who really know where rules are most likely to rub. To the Midwives' Institute, 12 Buckingham Street, Strand, belongs the honour of really drafting and pressing the Bill, and the needs of midwives have ever received consideration there. Through them, then, can any suggestion or queries be passed, and Miss Wilson and Miss Paget are both connected with the institute. Miss Oldham is connected with the Royal British Nurses' Association, and the members of that body should watch carefully the proceedings of the Central Board.

Next, a word of consolation to those who are frightened of the new Act. Though it has received the Royal Assent and is actually law now, its regulations do not come into force until 1905; so that midwives will have plenty of time to adapt themselves to new conditions. One new condition will be that no midwife will be allowed to use an uncertificated person as a substitute, and this will do away with the disgraceful practice of sending pupils unaccompanied to cases. The Act also protects the title of midwife in the following clause:—

"From and after the first day of April, one thousand nine hundred and five, any woman who not being certified under this Act shall take or use the name or title of midwife (either alone or in combination with any other word or words), or any name, title, addition, or description implying that she is certified under this Act, or is a person specially qualified to practise midwifery, or is recognised by law as a midwife, shall be liable on summary conviction to a fine not exceeding five pounds."

Finally, all midwives in return for the protection and status they are getting should be very careful of their own honour, to be in no hurry to grumble at regulations, and to do their best to put their profession on such a footing as to secure the respect of all their sex.

### Help the Nurses to Help the Sick.

**Royal National Pension Fund for Nurses,** 28 Finsbury Pavement, E.C.—The past year has again been one of remarkable success for this fund, the results in many cases surpassing those of 1901, "the record year." The policies issued during the year were over 800, the number showing no diminution as compared with the previous year. Upwards of £1,570 was distributed in sick pay, a fact which should especially appeal to nurses who are working on their own account. Over £7,550 was paid out in pensions and bonuses in 1902, an increase over the previous year of no less than £1,600. The premium income, *i.e.*, payment by, or for, nurses, exceeded £93,000, the total income for the year being £120,000. The invested funds of the society now stand at a figure considerably in excess of £700,000.

**The Junius S. Morgan Benevolent Fund** is an auxiliary to the Royal National Pension Fund for Nurses, and was founded through generous contributions from nurses themselves, and raised to handsome proportions by the munificence of the Morgan family and many other friends to nurses. The work is done by volunteers (many of whom are hospital matrons), under the supervision of an influential committee, which devotes time and care to the investigation of claims and the relief of urgent cases of distress amongst the policy-holders in the Pension Fund. Secretary, Mrs. Bretland Farmer.

**"The Hospital" Convalescent Fund.**—The object of this fund is to provide rest for weary workers amidst suitable surroundings, without any of that anxiety about ways and means which retards convalescence. Since the establishment of it many tired and delicate nurses have enjoyed a much-needed change of air such as they cound not possibly have secured for themselves without help. Experience has proved that it is better to let the nurses have a choice of locality rather than to send them to one settled place, and nurses are accordingly sent to all parts of the country. Contributions which would increase the field of usefulness are invited by the Hon. Secretary, care of the Editor of The Hospital.

**Queen Victoria's Jubilee Institute for Nurses.** Offices: St. Katharine's Precincts, Gloucester Gate, Regent's Park, N.W.—The Institute trains nurses in district nursing, and supplies nurses to affiliated associations for the sick poor in their own homes. Applications for information should be addressed to Miss Peter, the General Superintendent. Nursing associations in England, Scotland, Ireland, and Wales are affiliated with the Institute.

**East London Nursing Society.**—The object of this society is to nurse the sick poor in East London in their own homes by means of trained resident nurses, each nurse living in the parish in which she works. The extent of the society's useful work is shown by the fact that in 1901 the staff of 29 nurses attended to 4,966 persons, to whom 118,591 visits were made. Annual subscriptions and donations to the general fund are asked for. Secretary, Mr. Arthur W. Lacey, 43 Rutland Street, New Road, Commercial Road East, E.

**The Colonial Nursing Association,** the Imperial Institute, S.W.—This valuable association was founded six years ago to supply trained nurses to the Crown Colonies and small British communities in foreign countries. Since the foundation 144 nurses have been despatched to various parts of the world, grants in aid being made where it is clearly shown to be impossible for the residents unassisted to bear entire cost of passage moneys, salaries, and maintenance. It is one that appeals to the sympathies of all, for what family is there that has not some members in distant lands, building up the Empire, and fighting with the sickness that comes with rough faring and undrained country? The Hon. Secretary, Mrs. Debenham, will be glad to receive contributions, especially as an effort is being made just now to extend its benefits to the poorer colonies.

### Christmas in the Workhouse Infirmaries.

Although, as the report of the Departmental Committee appointed by the Local Government Board to inquire into the nursing of sick poor in workhouses shows, there is an enormous deal still to be done to bring poor-law infirmaries generally into anything like line with the great hospitals supported by voluntary offerings, substantial progress has been made during the last few years. Here, however, the question of training, of the supply of nurses, of the relations between superintendents and workhouse matrons, does not come under review. Indeed, every infirmary which is included in the series of contributions which follows this is a recognised training school, issuing its certificate for the three years' curriculum, and qualifying its probationers to fill any posts which may be open to them in the nursing world. The point, however, which it is desired to emphasise, and which is brought out in the clearest possible way, not only by the accounts of the Christmas festivities written on the spot, but also in the accompanying illustrations, is that the Christmas festival is now celebrated not less completely and enthusiastically in what one of our contributors calls our "great State hospitals," than in the institutions which, happily, are free alike from State aid and State control. How far this satisfactory condition of affairs is due to particular boards of guardians, or to particular medical and nursing staffs, it is not essential to inquire. Our contributors, some of whom, dealing with actual incidents last Christmas, write necessarily in the past, while others confine themselves to features which are usual on each occasion, justify the conviction that the excellent, and often generous, provision made for the sick and disabled poor in our workhouse infirmaries, is due to the efforts of all the authorities to do the utmost that lies in their power to render the Christmas season cheerful, homely, memorable, to every patient in the wards—from the child who can hardly toddle to the infirm old man or woman who has to be carried about. This is the spirit that should prevail, no less in hospitals which are supported by the rates than in buildings which are erected by the free gifts of the community, for the sick poor, whether they have been provident or improvident, are all alike in one respect. They have all an irresistible claim on the consideration of those who are charged with the care of them. It is a matter for much congratulation that the results of the splendid development of the hospital movement include not merely the utilisation of the resources of science and the most improved methods of nursing in the leading workhouse infirmaries, but also the wider cultivation of the humanising influences which, if exerted as a matter of course throughout the year, come into fullest play at Christmas. Our Plymouth correspondent mentions that the concerts, Christmas tree, and ward teas, were an innovation on the part of the new superintendent of nurses, last year, and though the season has been more or less joyously observed in some of the infirmaries for several years, there was a time, not far remote, when the unfortunate inmates had no reason to anticipate it with pleasure. Now, at least in the most important, there is the true hospital note, no less than the charming decorations, the good fare, the welcome gifts, the various entertainments that indicate the arrival of the period when all the world does its best to make merry—the note of sympathy for the suffering, which, whether it be evinced in tender concern for pain to be borne or in genuine interest for alleviation to be attempted, can do much towards investing the wards at Christmas with the attributes of a happy home.

### St. George's Infirmary, Fulham Road.

It would seem as though there could hardly be two greater extremes than the festivity usually associated with Christmas and the poverty and suffering sheltered in our workhouse infirmaries. Yet I think the Christmas season in these institutions, in spite of the sad remembrances it occasionally recalls, is a very happy, cheerful time to most of the sick people. There is no festival to observe in like manner during the summer time, and so it comes about that all our happenings are dated in relation to "last Christmas" or to "next Christmas." And, in a measure this is as it should be, for in a large community where many hundred persons are gathered under one roof and all who are able—from the staff in the great kitchen to the sufferers in the wards—are doing something extra to make Christmas a success, a spirit of exhilaration should pervade the place.

Each sick person does what he can to help with the decorations. Those who have the use of their fingers make wreaths of evergreens and artificial flowers, flags, and ornamental devices, also dress dolls for the children. Thus nurses and patients are both intensely busy until Christmas Eve, when the last preparations are made, and the familiar wards are charmingly transfigured.

Just before midnight on Christmas Eve Santa Claus goes round to every ward and leaves a token for each person. For the children these gifts usually take the form of large white muslin stockings embroidered with bright colours. Some toys go inside the stocking, but large as it is, it will not hold all. The stockings are put on each child's bed, and Christmas morning sees the children awake betimes! Their joy and pleasure are boundless. Toys that come in such number and variety are indeed appreciated.

Then follows the excitement of being specially dressed for dinner, and the enjoyment of unusual dainties at the feast. The Christmas dinner, even to the sick and ailing, is the important feature of the day, and for this to be successful great preparations are undertaken at least a month beforehand. It may be interesting to some to know the magnitude of the culinary preparations, so I briefly enumerate the quantities of fruit, etc., which have to be prepared for the plum puddings:—168 lbs. currants, 168 lbs. raisins, 70 lbs. suet, 180 lbs. flour, 6 lbs. sugar, 30 lbs candied peel, 320 eggs, etc., etc. The mixture is weighed out into puddings of 5 and 7 lbs. weight, and there are enough to go all round both on Christmas Day and New Year's Day.

The patients also have roast beef and roast potatoes, with lemonade, gingerade, coffee and cocoa to drink. Oranges, apples and cake for tea.

Everyone is more than contented with the dinner; even the very old and feeble delight in the little taste which is enough for them. The unusual bustle seems to revive them, as if they were themselves taking an active part in the day's pleasure. After dinner the men have pipes and tobacco and the women and children sweets and fruit. A great treat to the patients who are able to walk is the permission to visit other wards, so there is much calling on friends, with good-natured criticism of ward decorations to vary the afternoon's leisure. Those men who cannot leave their beds are allowed to smoke in the wards. This is a very great concession, and it is somewhat pathetic to watch the usually stolid faces relax into smiles over their pipes, all doing their best to be cheerful.

During the afternoon and evening the sisters and nurses entertain their charges in various fashions. The nationality or previous profession of the majority of the patients in a ward always gives it a special character of its own.

For many years the last entertainment of the day has been a concert held in a suitable ward where all the patients who are able to be moved are assembled and are entertained by the doctors and their friends with music, singing and recitations.

This finishes up a day, which everybody has enjoyed, by banishing as far as can be, for a few hours at least, pains, memories and disappointed hopes.

There are others connected with the Infirmary who enjoy their Christmas perhaps even more thoroughly than its inmates. These are the children, happily convalescent, who are temporarily boarded out at the seaside.

The following letter received from one of the elder girls speaks for itself. She wrote on behalf of 33 little ones, to each of whom had been sent a new sixpence received from *Truth*, and also a present. These coins from *Truth* and the toys are much appreciated, and some of the playthings may be seen in the accompanying photograph of a ward in which there are some children. In addition to these gifts the Guardians annually vote a sum of money for the purchase of toys, so that each child receives a suitable gift.

The Seaside.

"Dear Matron,—I now take the pleasure of writing you these few lines in thanking you for the big doll, and also the sixpences to go to the pantomime with. We went to see 'Puss in Boots.' We are going to have a play called 'Red Riding Hood.' Lizzie is a singing girl, and a market girl, and a fairy; Louie is Fairy Queen and I am Granny. We enjoyed ourselves on Christmas Day very much, we had a very big Christmas tree, and I had a lovely kid work-box, a pair of scissors, all coloured silk, cotton, and thimbles, three packets of needles, two reels of crocha cotton, a bodkin. And Bertha had the same; and in my stocking I had a silk neck-handkerchief. We had a lot of bon-bons, turkey and ham, Christmas pudding, mince-pies, oranges, and apples. After dinner we had a lot of games, some of us had our photographs taken before dinner. Matron is going to send you one of them when they come back. All the boys and girls send their love to you. We wish you a happy New Year. I must now conclude my letter as we are now in school doing our lessons, with much love from

[Signed] "A., B., C., D., E., etc."

But it is not only to the patients that Christmas is a notable season. All that is done for them involves great exertions from the nursing staff and the servants, and in the days following Christmas they in their turn enjoy a Christmas dinner and an entertainment, the latter frequently taking the form of the merriest dance imaginable. The quarters of the staff are happily situated far away from the sick wards, so that their festivity in no way interferes with the patients' rest. This being so, the merry-makers are able to enjoy their well-earned recreation without misgivings of of any kind.

A Female Ward, St. George's Infirmary, Fulham Road.

### Southwark Infirmary, East Dulwich Grove.

THE patients at Southwark Infirmary have a thoroughly enjoyable Christmastide. The wards are most charmingly and artistically decorated by the sisters and nurses who are unsparing of their efforts for the comfort and seasonable enjoyment of their patients. Varied designs are carried out in colour and decoration; art pottery, palms, ferns, and natural flowers, are arranged on centre tables gracefully draped with Liberty silks and art muslins; festoons of evergreens, flags, mottoes, fairy-lamps, and Chinese lanterns, are freely used with good taste and effect. Last year quite a realistic wintry scene was displayed in one ward, where a snow shower was represented by small flakes of cotton wool suspended from the ceiling by thread; this looked a most fairy-like scene when lit up with rose-coloured lights in the evening.

The festivities usually commence on Christmas Eve. At eventide the choir of St. Barnabas Church, Dulwich, accompanied by their vicar, who is also chaplain of the infirmary, sing carols in the corridors outside the various wards. These are greatly appreciated, and sound very soothing and sweet through the long corridors and wards where the lights are lowered, and the patients settling down for the night. The infirmary chapel is always tastefully decorated by the first assistant matron with white chrysanthemums, exotics, ferns, and evergreens. On Christmas Day celebrations of the Holy Communion are held at 6, 7, and 9, and (after morning prayer and anthem) at 11 A.M., so that as many as possible may have an opportunity to attend.

At 12 o'clock—dinner time—the wards are very busy, as the porters, with 'trolleys laden with Christmas, fare arrive from the kitchen. There are 789 beds in the infirmary, and a large resident staff, so cook has more than a few plum puddings to make. An abundant supply of prime roast beef, vegetables, plum pudding, and ginger beer, is provided every year by the guardians, followed by dessert of fruit, with biscuits, crackers, etc. On this one day in the year, dinner is allowed to be carved and served in the wards. As many of the patients as have permission from the doctors sit up for dinner, and greatly they enjoy the treat of dining at the tastefully decorated tables. Nor are those still seriously ill forgotten. On every "landing," one or more wards are reserved, and kept strictly quiet, for those too suffering to enjoy the festivities, where they are carefully tended by their nurses and special diet supplied for them.

A Women's Ward in Southwark Infirmary, showing the Snow Scene.

*From a Photograph by Mr. Lawrence, East Dulwich.*

After dinner the men who are able are allowed to smoke, tobacco and pipes being supplied by the guardians, and those patients strong enough to do so, go round the other wards to admire the decorations, and enjoy the songs, recitations, etc., going on all the afternoon and evening in the wards. The medical superintendent matron and medical officers also visit the wards from time to time. Many are the expressions of enjoyment and satisfaction from the patients, every one possible being cheered, from the guardians to the cook. In several wards pianos are permitted, and provided by the nursing staff; these add greatly to the liveliness of the scene, and last year one of the medical officers kindly amused the patients in many of the wards with a gramophone.

Tea is served at 5 P.M., cake and other good cheer provided for the patients gathered round the tables. As to the children, there are usually about 80 little ones of all ages in the infirmary wards on Christmas Day, including those in a large children's ward containing 30 cots.

Every child on Christmas morning receives a toy sent by *Truth*, and the elder ones a bright new sixpence each from the same kindly donor, and many other friends send toys, scrap books, dolls, etc.; but the great feature of the afternoon is the children's tea and Christmas Tree in the children's ward, the invitations to which are sent by the senior baby. As many of the staff as possible are present. Very sweet the little ones look dressed in pink frocks and muslin pinafores, the children from the other wards being carefully brought in by their nurses and other kind helpers. Those who are strong enough are soon seated round the little tea table, which sister has made to look so dainty, and the others are arranged comfortably in their cots. But, attractive as the tea table looks, it is quite eclipsed by the Christmas Tree of noble proportions which reaches from floor to ceiling and is generally a gift. Never was a tree so heavily laden with toys, and of course the customary fairy doll crowns the top. Tea over, the usual hubbub is resumed, and then the children gather round the tree. Great is the wonder and excitement of the little ones, as the medical superintendent, medical officers, and sisters commence to distribute the toys.

As the names are read from the labels attached to the toys, the eager children, utterly regardless of grammar, cry out, "Here I is," or "Him's me," pressing forward to receive the toys, which have been previously selected to suit their ages and requirements. Drums are beaten, trumpets blown, mechanical toys wound up, and dolls carefully nursed by the happy children, who appear to thoroughly enjoy their party; and sisters and nurses are to be seen busily looking after their little charges in the midst of the din and noise.

**A Women's General Ward in Southwark Infirmary.**

*From a Photograph by Mr. Lawrence, East Dulwich.*]

Last year, in the evening there was an entertainment given by Mr. A. Thomas, professional humorist, who greatly delighted the large audience of adults and children. The patients who were present retired when this was over to their respective wards, everyone agreeing that it was quite one of the happiest and brightest of Christmas Days. Lights were lowered and all quiet by 9 30 P.M.

During the last week of the old year concerts are held in several of the male wards, and in the women's wards. The staff are not forgotten. Christmas dinners of turkey, goose, mince pies, dessert, etc., are provided for them during the week, and extra leave is arranged for the nursing staff both on day and night duty, which is very much appreciated by them after their arduous and willing labours for the enjoyment of their patients.

### Bethnal Green Infirmary.

The decorations in the different wards at Bethnal Green Infirmary are very varied. In some, festoons and devices of evergreens and flowers are the order of the day, in others, flags are largely used, while others again confine themselves chiefly to goodly supplies of plants and flowers. In all, there are pretty coloured shades and artistic Japanese lanterns over the electric lights. These last are very decorative as they soften the lights and give a touch of colour which is most effective.

Last year the "Charity" children's ward was especially beautiful. It had been decorated by the Kyrle Society with a series of illustrations of nursery rhymes, and panels of wild flowers and scroll work, and these together with the white cots with flowered counterpanes, the little patients in their dainty coloured jackets, the bright eager little visitors, the well-filled Christmas tree, and the illuminated Japanese lanterns made a very attractive picture.

In the "Prudence" children's ward there was a large Christmas tree, and here a very large doll (sent with an assortment of toys by *Truth*) seated upon the ward rocking horse was a special source of pride to the children last year.

Nearly every ward is provided with a piano for the week.

On Christmas Eve the day sisters and nurses assemble at 10 P.M. and make a round of the corridors and landings, singing carols, the wards looking very pretty and artistic with their decorations showing dimly in the softly-shaded lights. The carol-singing on Christmas Eve is a favourite among the Christmas celebrations, both with patients and nurses. One old patient who was in the infirmary last year remarked a day or two before Christmas, that he "did not mind what was missed out if there was only the carol-singing again." The nursing staff, too, thoroughly enjoy the carols, and the hot coffee and biscuits served in the dining-room at midnight when the last post can be looked through, Christmas presents discussed, and congratulations on successful decorations exchanged, makes a pleasant homely gathering which all appreciate. Pleasant though it is, however, it is of necessity very short, as Christmas Day means long hours and a busy time.

A Children's Ward "Charity," in Bethnal Green Infirmary.

*From a Photograph by Messrs. Russell & Sons, Baker Street, W.*

On Christmas morning every patient receives a Christmas letter, and Santa Claus is busy among the children.

The Christmas dinner consists of roast beef, plum pudding, blancmange, jellies, fruit, and aërated waters, for those who can enjoy any or all of these good things; and after dinner pipes and tobacco are provided for the men and packets of sweets for the women. During the afternoon small parties of visitors give short entertainments, consisting of conjuring, singing, music, and recitations, in every ward except one or two where the patients are too ill to admit of any noise or excitement. After the entertainments comes tea, with cake and bonbons, and at 5 o'clock the "Charity" Christmas Tree is shorn of its fruit. All the children in the infirmary who are able attend at this function, and receive as many toys as they can carry. When the last treasure has been disposed of there are many sighs of regret and some persuasion needed before the little visitors can be coaxed back to their own wards.

### St. Pancras Infirmary, Highgate.

Christmas morning usually begins at St. Pancras Infirmary with the singing of carols by the nurses and probationers, and very picturesque they look coming down the long dim corridors, each one carrying a coloured lantern hanging from a gaily decorated pole. The patients much enjoy listening to the carols and watching the procession as it passes round their wards.

Breakfast over and the usual ward work finished, the morning passes very quickly. Little finishing touches have to be added here and there to the decorations, the tables prepared for dinner, and plants and flowers arranged on them to the best advantage. On Christmas Day dinner is served in the ward instead of in the day-room, so that the patients who cannot leave their beds may still be able to join in the Christmas dinner festivities.

Roast meat, parsnips, potatoes, plum pudding, lemonade, ginger ale and soda water are provided for all who are able to partake of Christmas fare. For those who are not, there is a choice of chicken, sole, and custard or other milk pudding. Pipes and tobacco are supplied for the male patients and sweets for the women and children. Last year, one of the committee kindly sent oranges for dessert. Nearly all were able to heartily enjoy their Christmas, and while in the female wards the women amused themselves with crackers and sweets, the men had an after-dinner smoke. One of the privileges of Christmas time which is the most appreciated is that in the male wards the men have permission to smoke on Christmas, Boxing, and New Year's days.

At two o'clock all patients who are sufficiently well are allowed to make a round of the wards to see the decorations, and compare them favourably or unfavourably with those of their own special ward. This part of the day is always much enjoyed and takes about two hours, all returning to their respective wards in good time for tea. The wards look very gay and bright with flowers and plants, wreaths of evergreens, all sorts of paper flowers, flags, and mottoes. The preparation of these decorations gives great pleasure and interest to the patients for some weeks previously, and many of the flowers and mottoes are made with singular taste and skill. It is really wonderful to see the variety of decoration can be done with the same colours and materials—no two of the wards ever look alike. Some artists prefer long drooping wreaths of evergreens hanging down from the ceiling across the room, others have festoons of paper roses, with little or no evergreen, dependent from the middle of the beams of the ceiling to the sides of the walls. In some wards there are groups of flags, and a variety of mottoes, and all the hanging baskets and pots and the flower-stands are filled with ferns, grasses, and flowers. One ward last year had broad bands of green paper, lined with daffodil yellow, twisted from the centre of the ceiling beams, carried loosely down and fixed on each side of the wall a little above the head of the beds. This ward was all done in the same shade of green and yellow. The children's ward, too, was very prettily draped with festoons of poppy-red tied up with white bows to the picture rods, looking like a gay frieze on the pale

A Female Ward, St. Pancras Infirmary.

ST. PANCRAS INFIRMARY, HIGHGATE—*Continued.*

green walls; wreaths of hops and baskets of flowers in the middle of the ward, contrasting with the red-draped cots and white counterpanes, made a very pretty picture. The little ones are always a special object of interest to the adult patients.

On Christmas Eve last year Mr. and Mrs. Regnart, of Highgate Lodge, sent to the children as a Christmas present a large beautiful rocking donkey, a pair of grey rocking horses, and a piebald riding pony; and these, with the group of lioness and cubs and the elephant (the gift of Mr. and Mrs. Regnart on Christmas, 1900), were great sources of interest and amusement during the afternoon. Mrs. Regnart also supplied the Christmas stockings for the children's ward and for all the other wards. The Santa Claus Society sent their usual liberal provision of toys, cards, sweets and books for the filling of the children's stockings. On Christmas morning the elder children have *Truth's* new sixpences, and on Boxing Day each child chooses a toy from *Truth's* box of toys, games, dolls and crackers. Last year *Truth* also sent a large Punch and Judy Show, and we were fortunate enough to have in the infirmary a patient who was a show man, so that he was able to give some very clever sketches and songs in several of the wards. The evening programme is necessarily a very quiet one, but the patients are allowed to visit from ward to ward on their own blocks, and with the help of the staff get up some very bright and pleasant little concerts. Recitations, comic, patriotic and sentimental songs make a very happy evening's entertainment. At nine o'clock all patients are in bed, and, though tired, everyone agrees that they have spent a "very quiet but happy Christmas Day."

### Birmingham Infirmary.

I AM always glad of an opportunity of bringing forward the way in which Christmas and other festivals are recognised in our great State Hospitals. The public in general has no idea of what merry and bright times can be spent then by the sick and disabled poor. Christmas Day itself presents a most cheerful and homely aspect in the Birmingham Infirmary. All the wards are decorated most daintily with garlands of evergreens, suitable mottoes, Japanese lanterns, umbrellas, and pretty and varied drapery. Drapery in a hospital ward does not, of course, sound very correct, but it is not left up long, and is of a very light description.

The wards here lend themselves very easily to decoration, they are so lofty and long that the garlands of evergreens strung across from side to side have the most pleasing effect, and paper umbrellas, shading the gas lights, look really very pretty.

The Board of Guardians for this city are most kind in the matter of presents, and every child under 15 years wakes up to find Santa Claus has left a well-filled stocking lying on the bed, with apples, oranges, sweets, toys, necklets, etc., contained therein. Also these same lucky children each have a parcel containing two really nice presents.

Every grannie in the infirm wards has a present in the shape of a work-box, a scent and soap box, a hand-glass—for old ladies have plenty of vanity left—or something of this kind, which greatly delights their dear old hearts, and make them feel that though old and helpless, there is someone who remembers them at Christmastime. The old gentlemen do not care so much for these fancy articles, so they are made happy with plenty of tobacco and snuff. Then, if any old lady cares for either of these commodities she may have them too, but there are not many who do.

The day begins very early in the morning, when carols are sung by members of the nursing staff in the main corridors.

The patients' dinner begins at 11.30, after service in church is over, and it is interesting to see how daintily some of the sisters arrange the tables for their convalescent patients' dinner—pretty flowers and pots deck the usually rather severe board, and crackers are laid by the side of each knife and fork. Last year there was much merriment, for some of the more ignorant patients had never seen crackers before, and thought them not safe. One old lady amused us very much by eating hers, and saying, she found it "rather tough and not very tasty," while another "thought she had eaten enough for to-day, and would save that for to-morrow."

The dinner itself consists of roast beef, roast pork, and good plum pudding. There is no limit to either, and the number of times plates are replenished, more especially among the men, is really most astonishing. However, if fare is good, it does not do much harm, and very few seem any the worse next day.

Those unable to enjoy the good fare are provided with jelly, custard, grapes, etc., that they may not feel left out.

There is general liberty all day, and patients walk about and smoke their pipes as though they were at home.

A band often plays cheerful selections of music all the morning, where the people are not too ill to stand a noise, and in the afternoon there are impromptu concerts in several of the wards, which call forth dormant talent most unexpectedly in some of the patients.

Several of the sisters provide cake, jam, etc., for the patients' tea, and apples, oranges, sweets and nuts are left standing about for them to help themselves all day.

The evening is spent in visiting about and listening to music in the various wards; everything generally passes off quietly and well, and the consensus of opinion among the inmates of the whole infirmary at the end of the day is that they have had a very happy Christmas.

For more than a week following entertainments are held daily in the various wards.

Boxing Day begins with a real old-fashioned party in a children's ward. The fun commences with a tea and games, next comes a Punch and Judy show in quite old style, which causes much merriment among the older children, though the little ones often fail to understand so much hitting about as Mr. Punch gives and receives. After this show, a most elaborate tree is lighted up, upon which are presents for all. It is quite pathetic to see the keenness of the little sick babies to be carried up to receive their own presents in person. Later come excellent concerts, got up by friends to which a great many convalescent patients from other wards are invited. The chief feature of this entertainment last year was the singing of Welsh songs by a Welsh choir, which was really quite charming. On the same day the female epileptics had a Christmas Tree and a happy evening of games and dancing. The following day the male epileptic patients had their Christmas Tree, each having a present suitable for his own use, accompanied by a pipe and tobacco. After the presentations a concert and magic lantern entertainment was given by the friends of one of the Guardians. Three days later there was again a concert in one of the men's wards, when amongst other items a most beautiful quartette from the Edgbaston Oratory sang in a manner which won the hearts of all the listeners. After concerts had been held in nearly all the other wards a day was selected for our own nurses' entertainment, which is looked forward to by patients and all inmates with the greatest possible interest. In 1900 the nursing staff gave a most excellent show of Mrs. Jarley's Waxworks and other items; but last year the programme took a quite original form. The nurses had been busy planning and arranging the details in their off-duty time for months, and by the enthusiasm with which they were applauded they must indeed have felt that no effort had been in vain. The programme commenced with a "Toy Shop." About eighteen nurses had dressed up representing all kinds of mechanical toys; waltzing, dancing, jumping, talking dolls, animals, Jack-in-boxes, a clown, a baby, a jumping coon, and several others, not forgetting tin soldiers, and a dancing bear. The shop was presided over by a "Spirit of Toyland," in an old-world dress, bespangled with toys and with birds nestling in her hair. Other principal characters were a mother and child, presumably come to purchase toys, but the bewildering scene and the great interest in the workings of all the objects rather interfered with the purchasing. The text of the piece had all been composed by members of the nursing staff, together with the conception of the whole performance. Other items on the programme were a duologue, "Cheerful and Musical," in which the character of a would-be "lady's companion" was most ably played by one of the sisters. The second part commenced with an adaptation from the "Geisha" entitled "Happy Japan," in which about twenty nurses, dressed mostly in Japanese costumes, sang, danced, and tripped about in a most fascinating Japanese style. One of the nurses sang sweetly the solos of "O Mimosa San;" and the character of Wuni, the Chinese keeper of a tea-house, was a wonderful piece of ingenuity on the part of one of the sisters. A set of charming tableaux vivants, and suitable music, together with a selection of coon songs by the coon chorus, concluded the programme. The delighted audience showed their appreciation in a most hearty manner, and the matron was earnestly entreated to have the whole performance repeated on two other occasions, so that every patient who was well enough, and every member of the staff might have an opportunity of seeing it.

An Infirm Ward in Birmingham Infirmary.

The three following evenings of the week were filled up with ward concerts, and so the Christmas festival ended. The illustration will give some idea of how one of our wards looked in its Christmas dress, but no photograph can show the joy and contentment which seemed to pervade this institution during the whole time, nor the happy memories which must ever remain of "Christmas in the Birmingham Infirmary."

### Leeds Infirmary.

"'Tis a kindly open-hearted Yule, we cherish most the memory of friends."

In a large institution with nearly 730 inmates, there are, no doubt, many whose thoughts revert to "home" at Christmastide, let the vicissitudes of their life be what they may and home "ever so humble." The nursing staff of Leeds Infirmary employ every effort to render the surroundings of the patients bright and cheerful. For weeks preceding Christmas the annual carol singing "practice" commences, and the different designs for ward decorations are got well

LEEDS INFIRMARY—*Continued.*

in hand, each sister trying her utmost to make the wards look pretty. On Christmas Eve a choir formed of the day nurses make a concerted tour of the wards, assisted by several friends who offer their services to accompany with their violins. The carols are generally well rendered, and are much appreciated, especially by the old people. On Christmas morning the children from the union schools come round and carol to the patients, afterwards paying a visit to the Nurses' Home, to cheer the matron and residents there. This band of young voices is headed by the master of the schools, under whose able management and training they sing most heartily. The patients' dinner is the next important item on the programme. Much care is exercised by the medical superintendent regarding the special dietary membered by the chairman of the Board, inasmuch as a large, beautifully-iced cake was received by the matron "with the chairman's hearty greetings for the residents of the nurses' home." The thought and gift were much appreciated. The great event of the year comes off early in January—the nurses' annual dance—to which everyone looks forward. Last year it was held in a large room at the schools away from the infirmary, which was very tastefully decorated. It is quite a reunion, as the old probationers and nurses who have left are generally invited. Nothing is spared to make everything quite a success, and the nurses come two evenings, so that the wards remain well staffed. The Chairman of the Infirmary Committee very kindly gave the nurses their treat, and the pleasure which beamed on his face at

A Ward in Leeds Infirmary.

for the patients, each having what is considered best for him. The usual roast beef and plum pudding are prominent on the bill of fare, and many dainty dishes are provided for those more seriously ill. The wards are visited during the day by several members of the Board and their friends, who seem pleased to see the patients so comfortable and responding to the hearty Yorkshire greeting, "Hi! wilt tha' coom hinny and have a teaste; it's rare stoof!" During the evening the patients are entertained by the staff in various ways, the honoured chief, "Father Christmas," causing much diversion. In the children's ward the Christmas tree is laden with gifts for the little ones—and when Father Christmas appears their "great expectations" are fully realised. Last year the staff were really individually re- his being the means of affording so much happiness to others will be long remembered, and he expressed himself delighted to see the young folks enjoy themselves. At the close of the dances the medical superintendent, in a few well-chosen remarks, pointed out the advisability of the nursing staff in their surroundings being bright and cheerful, but in dealing with the sick and suffering never to forget the one end and aim of their work. After the dance the festivities are supposed to be at an end, and each nurse is expected to settle to work again, and prepare for the final examination which comes off in May. And in this again the nurses have encouragement inasmuch that they have prizes awarded to them for those highest in order of merit.

### Cardiff Union Hospital.

At the Cardiff Union Hospital preparations for the coming event are begun during November, when old garments and old bed covers are as far as possible replaced by new, so as to have plenty which are fresh and bright in use on Christmas Day. With December come the preliminaries to the decorations, quantities of really beautiful paper flowers made by the nurses, and mottoes, etc., all got in readiness. The arrival of the evergreens is the signal for putting up the decorations, and the wards look exceedingly gay and pretty when finished. In addition to the toys given by the Guardians, kind friends also send gifts, so that the children, of whom we have an unusually large number, have a very happy time. The heavy toys are given to them on Christmas morning and the lighter ones are put on the tree, which is stripped for their benefit on New Year's Day. Nor is the nursing staff forgotten—cakes and bon-bons often arrive for them also. Breakfast is always served to the patients half an hour earlier than usual on Christmas morning, and as the superintendent of nurses makes her first round she distributes to every adult patient "A Christmas Letter for You." Services, both Church of England and Roman Catholic, are held in the chapel, and usually well attended. About noon the great event of the day, dinner, is served. Wherever possible each charge nurse has all her patients together in one ward, so that she can see that the individual needs receive proper attention. There is much friendly competition among the nurses to beautify the tables, which are decorated with plants and cut flowers, the apples and oranges being also arranged with a view to effect as well as convenience. The dinner consists of roast beef, potatoes, vegetables, pudding, apples, oranges, and either beer, tea, coffee, or lemonade, and for the men tobacco and the women and children sweets. Christmas fare is general on that day, milk being the only alternative diet. Last year we were fortunate in not having any patients who could not take some of the good things provided. Several of the Guardians visit the wards during the dinner hour, their congratulations, kindly inquiries, and jokes helping to brighten the already animated scene. The superintendent of nurses is spared one great anxiety at Cardiff, namely, the cooking of the patients' dinner. The hospital, though a separate building, is in the grounds of the workhouse, so that the dinner is sent from the workhouse kitchen ready cooked and all she has to do is to see that each charge nurse has a share in proportion to the number of her patients, in which duty the resident medical officer helps. As far as beef and pudding are concerned, the only limit is the patients' appetites, for though a rough estimate is made as a guide to primary distribution, a request for "more" receives prompt attention.

A Ward in Cardiff Infirmary.

At 2 o'clock the nurses have their dinner of turkey, pudding, pies, and dessert, the table being decorated with white flowers, holly and mistletoe.

Visiting is the general amusement during the afternoon. No member of the staff is allowed to go outside the boundaries, but duty hours are not adhered to, and the restrictions as to visiting other parts of the workhouse are

CARDIFF UNION HOSPITAL—*Continued.*

removed that day for the inmates and patients as well as for the staff.

Between four and five afternoon tea is served for the nurses in the superintendent's room, the little rest being very welcome, but at five all go to their own wards for the patients' tea, after which anyone who possesses a musical instrument brings it out. Between us, last year, we had two mandolines, two guitars, and a banjo, and so were thus able to accompany songs and also to have a very little dancing and some quiet games in the day-rooms until eight. At that hour the patients retired for the night, and soon after the nurses had their supper. Later on those nurses on the day staff who felt able joined other officers of the workhouse in the inmates' dining-hall and danced for an hour, finishing with "Sir Roger." We separated at 10.30 feeling that we had had a most successful day. During January and February some of the musical societies promise to give concerts in the inmates' dining-hall.

### Plymouth Infirmary.

Last year the festivities at Plymouth Workhouse Infirmary began for the first time on Christmas Eve with a musical evening, supper, and dance, given by the superintendent to the nursing staff and their friends, which proved a great success. No pains were spared to make the meeting a most enjoyable one. At the close of the entertainment a party of forty ladies and gentlemen arrived, and being joined by the superintendent and nurses, sang carols in the various corridors, the ward doors being left open so that all the patients could hear and join in. On Christmas Day many good things are provided for the patients able to partake of them, both by the Guardians and other kind friends interested in the infirmary. The dinner consists of roast beef, vegetables, plum pudding, fruit, etc. All the male patients are supplied with tobacco, and have permission to smoke any time they choose throughout the day. During the afternoon the wards are thrown open, and the convalescent patients allowed to visit each other. The rest of the day is spent in story, song, and sentiment, and many are the amusing and interesting contributions by the jolly Jack Tars and old soldiers, some of the latter "yarning" about "South Africa," whence naturally some have recently returned. At 7 30 all retire, well pleased with what has been provided for them, and often many of the patients say that they have never spent such a happy Christmas before. The superintendent and nurses dine late, and after dinner are usually joined by a few friends, music being kept up till midnight, refreshments served at 10.30. During the following week teas, concerts, and entertainments are given in most of the wards. The children's tea and Christmas tree are given about December 30th, and a Punch and Judy show often

A Surgical Ward in Plymouth Infirmary.

follows, which gives much pleasure to the little folks. The decorations are most elaborate, and some of the designs really artistic. Mottoes of all kinds adorn the walls, flags of every nation are to be seen, from the ceiling hang Chinese lanterns, coloured chain festoons, trailing ivy and evergreens. Pretty art muslin curtains drape the windows, whilst the mantelpieces and window bottoms are covered with white cotton wool (which looks like flakes of snow) intermingled with green leaves. There is a good supply of plants and flowers, and when the wards are lit up they are most effective. Very great praise is due to the nursing staff, ward assistants, and patients for the great taste displayed in the decorations; one and all working most willingly. The festivities, which are kept up for a fortnight, generally conclude with a concert.

### Stapleton Workhouse Infirmary, Bristol.

Festivity and jollity with those stricken with illness so serious as to necessitate their spending their Christmas in a workhouse infirmary obviously cannot have full play, but the predominant note of cheerfulness which characterises the wards we find one devoted to the Army, another the Navy, a third dressed rigging fashion, others equally pretty but with no special feature. The female wards are also extremely tasteful, dressed with different colours; amongst the

The Nurses of Bristol Infirmary in Fancy Dress.

*From a Photograph by Mr. Lindon Hatt, Clifton.*

adornment of wards must have its good effect even on those racked with pain. This remark can be applied with peculiar emphasis to the Stapleton Workhouse Infirmary at Bristol. In the main entrance of the establishment is a large motto, "The Staff of Life on the Point of Death," depicted by affixing small loaves to the points of several swords. The large day or sitting-room in which the convalescent patients of both sexes gather during this festive season is prettily decorated with evergreens and mottoes, to please all. The ornamentation of the wards and corridors is graceful and artistic. The season's greetings and good wishes are effectively arranged, and worked out in capital designs and with various backgrounds and trimmings. Amongst the male evergreens are numerous fairy lights and coloured lanterns which add a charm to the already pretty scene. The joviality of Christmas Day begins as early as 5 o'clock in the morning, when the nurses make the round of the wards singing carols, their pleasing vocalisation being everywhere greatly appreciated. The Christmas letter is a special pleasure to those who have never before spent a festival in hospital, and many are the surmises as to its sender.

Breakfast is served at 7.30 as usual, then for a little while the ward work takes place.

Lunch, which includes coffee, lemonade, or beer, is served about 11 A.M., after which there is more carol singing until dinner time at 1 o'clock. This important meal consists of the

**STAPLETON WORKHOUSE INFIRMARY, BRISTOL**—*Continued.*

time-honoured fare of roast beef and plum pudding. Dinner over, each male patient is supplied with tobacco and oranges. Snuff takes the place of tobacco with the female patients.

During the afternoon the patients are taken by the nurse of the ward through the other wards of the hospital, and many and varied are the remarks passed upon the decorations during their tour.

Tea is given at 5 30 P.M., consisting of bread and butter and cake *ad lib.* At intervals during the day songs are rendered by nurses and patients in most of the wards, and a day of real pleasure is brought to a close with a waxwork exhibition admirably arranged by the superintendent nurse. This is only a brief outline of the day's proceedings. During the whole week various kinds of amusements are got up for the benefit of the patients, such as character-sketches by the nurses, magic lantern entertainments by the chaplain, together with songs and recitations of all descriptions. The inmates enter heartily into all the rejoicings and duly appreciate the efforts made to provide them with good cheer. The Guardians, including their chairman of the Board and Hospital Committee, visit the wards during Christmas Day and express gratification that so much pleasure has been given to those who from force of circumstances are away from home at Christmas time. The superintendent nurse and her staff who work so loyally and energetically in their endeavour to brighten the lives and cheer the hearts of those in their charge, must feel somewhat repaid when they see how their endeavours are met with success and appreciation.

### Crumpsall Infirmary, Manchester.

CHRISTMAS in a large workhouse infirmary is much like Christmas in any other large hospital, only "rather more so," owing to the number of patients—nearly 1,200—to be provided for. Preparations begin some time beforehand, and the long ward tables are gay with flowers and berried holly. The well-polished floors and the white quilts with their scarlet borders help to give the wards a very smart and pleasing appearance.

A Children's Ward in Crumpsall Infirmary.

and nurses vie with each other as to ward ornamentation. Large bundles of evergreens are sent in, and the usual wreaths and mottoes adorn the walls. There are 42 wards at Crumpsall, each containing 31 beds, so that a large amount of decoration is required to make any appreciable show. The doorways are festooned with pretty curtains of art muslin, or decorated with trellis work covered with ivy,

In all the children's wards the day begins with distribution of toys, which are put into each child's stocking the last thing on Christmas Eve by the nurses. The wards are soon filled with musical (?) sounds, owing to the number of drums, trumpets, and mouth-organs provided. There are also all kinds of dolls and toys to suit the various tastes, from the baby just old enough to appreciate a rattle to the girl "almost too big for dolls," and the boy developing a taste for miniature carpenter's tools. In the adult wards the first hours of the morning are comparatively quiet, but in all the great excitement of the middle of the day is the dinner, which consists of the usual Christmas fare of roast beef and plum-pudding, for those who are well enough to eat it. As each ward sister has 62 beds under her charge, the serving of the dinners is a work of time; the eating of them is also a longer business than on ordinary days. The nurses, having seen the patients' dinners served and eaten, are ready to go down to their own excellent Christmas turkey, plum-pudding, mince pies, and dessert. This is served in the nurses' large dining-room, which is prettily decorated, and the table arranged with plants and flowers. As only half the nurses can be spared from the wards at a time, the first dinner is at 12, the second at 1. The night nurses, who have gone to bed early, are allowed to get up at 4 and join the day nurses in the wards for tea. Their dinner takes place at 8 P.M., and is an exact repetition of the day nurses'. The matron presides at all three nurses' dinners, and serves the turkeys and plum-puddings with the help of her assistants.

The afternoon is given up to visiting. All nurses and convalescent patients are allowed to visit any wards they like, and they go from ward to ward, comparing the decorations with their own, and speculating as to whether the Chinese lanterns in one ward are equal to the paper chains in another, etc. The matron also visits each ward, to admire the effect of the finished decorations, hear how each patient has enjoyed the Christmas dinner, and look at each of the children's toys. Tea and plumcake, or "currant bread" as the patients call it, follows at 5 o'clock, and at six a concert is given in one of the men's surgical wards, to which all patients who are well enough are allowed to go. The performers are the nurses and resident doctors, and a varied collection of songs and choruses fill up the time pleasantly till a quarter to eight, when, after heartily joining in "God save the King," the audience retire to bed, and another Christmas Day is successfully over.

The Christmas festivities, however, do not end with Christmas Day. During the first month of the new year various entertainments take place in different wards. These are generally little concerts, given by the nurses and their friends, and lasting from 6 to 8 P.M. First comes the Christmas Tree in one of the children's wards on New Year's Day. A large tree in the centre of the ward is gaily decorated with dolls and toys of all descriptions. After an early tea, which the children can hardly eat for excitement, the tree is lighted up, and the convalescent children from other children's wards are brought in, and all nurses who can be spared from the wards come to see the fun. The doctors come in dressed in quaint costumes, at which the smaller children hardly know whether to laugh or be frightened. However, they are soon persuaded to think it great fun, and to forget their fears in delight, as the doctors and nurses cut off and distribute the presents. Every child has a toy and a bag of sweets, and soon the pretty tree is stripped and bare again. Then games are played for another hour, which the doctors and nurses enjoy quite as much as the little patients; after which the children are carried off again to their respective wards, with their toys clasped in their sleepy little arms.

One of the best entertainments last year was given in a women's ward for chronic cases, whose beds and wheel-chairs were put in the front row of spectators. The first part consisted of songs and recitations given by the nurses. "The Three Old Maids of Lee," "Three Little Maids from School," and the dialogue of "Them Geese," caused special delight, as the performers were dressed in character. Then the resident doctors acted "Box and Cox," and great amusement was caused among the patients by the discovery that the doctor of their ward was taking the part of Mrs. Bouncer. Many amusing remarks were heard, especially on the way in which Mrs. Bouncer made Messrs. Box and Cox's bed, leaving the sheets hanging down at the corners and the quilt awry. Sister would have something to say if beds were made that way in her ward, and they thought Mrs. Bouncer hadn't had much experience in that work.

Thus in various ways, and thanks to the keen personal interest taken by the medical and nursing staff in the Christmas festivities, all patients, young and old, who are able to enjoy the fun, get their share of pleasure, and the Christmas season is looked back on by all as the brightest and happiest time of the year.

### Liverpool Infirmary.

Christmas Day in the infirmary of the Liverpool Workhouse begins at about three in the morning, when the night nurses go round the wards, lay a Christmas letter and card on the pillow of every patient, and a bag of sweets for every old lady in the female infirm wards—the old men getting their present of tobacco later in the day. At half-past five a large party of the day nurses begin their day by singing Christmas carols under the windows of the nurses' home, and then in as many of the wards as they have time for before their seven o'clock breakfast. The patients enjoy this very much.

The wards have all been decorated beforehand with evergreens, plants, lanterns, flags, etc., and look very festive and bright.

There is service in the church at 9 A.M., which all the patients who are capable attend, and as many of the nurses as can be spared. During the morning the visiting staff and many of the Guardians go round and wish the patients a merry Christmas.

Then comes the much-looked-forward-to Christmas dinner, when all who are allowed it have roast beef and mashed potatoes, plum pudding, coffee and fruit, and looking at the recipients of the good fare, one is left in little doubt as to whether they are enjoying themselves.

During the afternoon, the patients may visit their friends in other wards, and the men may smoke all day without fear of breaking rules. In the evening, small informal concerts and entertainments are got up in several of the larger wards, and the nurses do their utmost for the pleasure and comfort of everyone.

The children have their own especial Christmas treat a few days later—which consists of a Christmas tree and a Punch and Judy show. The tree is placed in the centre of a large ward and nearly touches the ceiling, and when all the candles are lighted the children get very excited and enthusiastic. Some are unable to leave their cots, but many are well enough to sit up and be dressed, and all seem to enjoy it thoroughly, though just at first some of them are too much awed to do more than gaze with mouth and eyes both wide open. After they have admired it sufficiently, the toys are distributed, and as each child's name is attached to its present there is no danger of any-one getting more than their share. Toys are also sent to the children in the whooping-cough, measles, and chicken-

**LIVERPOOL INFIRMARY**—*Continued.*

pox wards, because, of course, they cannot be present. The Punch and Judy show is always much appreciated, and by the time that is over the children are quite ready for bed, and all go off very happy and excited. The dolls on the tree are the result of an annual doll-dressing competition, in which some 60 of the nurses and probationers take part. The clothing of many of the dolls is beautifully made, and in some cases a good deal of imagination is shown. A local Sunday School, too, each year sends a deputation with toys for the children, usually a week or two before Christmas.

The nurses also have their Christmas dinner of turkey, goose, plum pudding, mince pies and fruit—but perhaps the chief event of the day for them is the arrival of the post, which brings them huge piles of letters, cards and parcels. They have an annual entertainment which takes place in the nurses' home in January. Sometimes the nurses and probationers combine and give a joint performance, and other years they give separate entertainments, in which case there is a good deal of friendly rivalry. The proceedings usually consist of a concert followed by either tableaux vivants or a dramatic sketch with an interval for conversation and refreshments. The amount of musical and dramatic talent at the disposal of the managing committee naturally varies from year to year. There is a movable stage with electric footlights, and the programmes, which are hand-written and painted from original designs by some of the nurses, make interesting little souvenirs.

### Portsmouth Infirmary.

It is written "Side by side with the Christmas 'art of giving' may be ranked the 'art of amusing,' which, like its sister, is a fine art, imperfectly studied and greatly in want of invention, variety and general re-study."

This sweeping reflection on most people's capabilities of amusing others can, however, hardly be applied to the Portsmouth Parish Infirmary, where the staff, certainly with very little study, get up really good entertainments for the patients with much vigour, ingenuity and considerable novelty.

It is generally arranged that the last lecture is well over before Christmas, so that the nurses with easy minds are free to give their time to decoration, amusement, and other frivolities. In an incredibly short time pretty finishings of art-shaded paper and muslin are made, the work of some proving that their hands are not new to it. Portsmouth, fortunately, does not suffer, as do many large towns, from a lack of materials. There are plenty of evergreens, and even bright holly berries to be had, and all the wards have as much as they want or can use, while the Children's Hospital has a fine tree.

The decorations differ in every part and are a great source of fun and rivalry. Last year "the palm" was supposed to be carried off by the nurse in charge of the approach to the doctors' quarters, where the evergreens were wreathed into a perfect bower, lit up by many brilliant coloured lanterns; but each nurse rightly held that her own ward was the nicest, and indeed it was hard to choose. Entertainments are generally held about the 27th and 31st of December, and are very enjoyable. The idea aimed at is to get everybody to do a little towards the amusements, and also to arrange so that all the patients shall have some of the enjoyment.

Last year Mrs. Jarley's Waxworks delighted the audience and were an immense success, about 26 nurses taking part in them. Mrs. Jarley, herself a staff nurse and a very clever actress, was helped by John, the ever-ready assistant dispenser, who "lifted" the figures and "wound them up." There was "Queen Eleanor," resplendent in a scarlet robe bordered with ermine, with a gold crown, sceptre, and orb, looking like a glimpse of the Coronation; a "Maiden all Forlorn," "Rosamond," "Rebecca and Rowena," and Cupid managing to shoot a cabbage for a heart. One of the best figures was Medusa. It was strikingly good and thoroughly realistic, snakes being cleverly made out of paper. Then there was our old friend "Bluebeard" and half-a-dozen of his wives. The curtains on the temporary stage set up in the wards were so arranged that not only did "Bluebeard" do his customary murderous deeds in beheading all his wives, but the heads of the poor ladies were shown afterwards hanging up by their hair, nodding and gory. Another good figure was Mrs. Bardell, who shed tears on being "wound up." The "Babes in the Wood," two nurses in white baby dresses and caps, sitting in wooden tubs, gained much applause, as did a particularly well got-up "Japanese dancing lady," a Joan of Arc, tied to the stake, and Beatrice with her turbaned head. All the waxworks, when duly wound up by "John," did the usual jerky movements peculiar to their kind, and caused roars of laughter among the lookers on. The "Old Nurse and the New" must not be forgotten; the old nurse was of the Sairey Gamp style, dirty, untidy, and not too sober, and the new was a sweet "Red Cross" nurse in white. The tableaux were interspersed with songs. The Pierettes vied in popularity with the waxworks. Ten girls dressed in white, with black pompoms, only relieved by the pretty many-coloured sets of ribbons attached to their mandolines, made up the troupe, and despite a little becoming nervousness and shyness, the music went well. A good dinner on Christmas Day was much enjoyed by the patients, many guardians and friends escorting the Mayor and Mayoress round the wards and kitchen during the meal. A good dinner and plentiful dessert on Boxing Day were well earned by the staff; a social evening later among the nurses being not the least enjoyable of the festive doings. All these Christmas doings had the hearty co-operation and sanction of all the head officials, and the entertainments were graced by the presence of the chairman of the infirmary committee and his wife, with many other guardians and friends.

I must not forget to add that nearly all the toys, of which there were many, were sent by "Uncle Taff," of the *Portsmouth Times*, from his little nieces and nephews, the children of the "League of Love," and that he remembers this place most handsomely every year. Mrs. Dupree, the Mayoress, when going round the children's hospital, kindly distributed some of these toys, and the Mayor gave each child a new sixpence.

One sweet little incident must be mentioned. An old lady in the infirm wards, belonging to the class of the old-fashioned gentlewoman, now fast dying out, in July, 1900, had been introduced to the Duchess of York; when she went over the infirmary last year, the granny worked the "dear" Princess of Wales, as she respectfully called her, a sampler or two and a few penwipers, and with the graciousness of our Royal Family, the Princess of Wales accepted the humble gift, and sent the old lady, through the medical superintendent, a note and a Christmas card. The patient now treasures her card among her most valued belongings, and is more than ever devoted to the throne.

### Christmas Books.

No Christmas present is more acceptable than a good book, nor is it always easy to lay one's hand on the right thing. In order to assist our readers we have classified the various books and publications which have come to our notice, so that the difficulty of selection may be, at any rate partially, overcome.

### BOOKS FOR LITTLE CHILDREN.

Thomas Nelson and Sons have contributed largely to the nursery library this Christmas. "The Friend of Little Children" (3s. 6d.) is a short Life of Christ, beautifully illustrated by Mr. John Lawson, and printed in large type. In "Golden Gleanings" (6d.) one finds many of the familiar Bible stories adapted for the youngest of readers, and illustrated in simple taste. Pictures replace words most effectively in "The Prince of Peace" (1s.), and we have the early history of Jesus told by a series of excellent plates. "Country Cousins" (3d.) and "Pick-a-Back" (3d.) are pretty little nursery stories, illustrated, and told in rhyme; "The House that Jack Built" (3d.) falls within the same category, whilst "Can't You Talk" (6d.) is similar, but on a larger scale.

The Doll Man's Gift. By Harry A. James. (George Newnes, Limited. 1s. 6d.)

This is a fairy tale, really beautifully illustrated by K. M, Skeaping. The evolution of an apple pip is cleverly told in a fairy-tale way, and the book is full of adventure.

The Admiral and I. By H. Escott Inman. (Ward, Lock and Co. 3s. 6d.)

Few books would be better suited for reading aloud to a young audience than this partly naval and entirely militant fairy book of Mr. Inman's. The tournament or duel between Ugly Muggy and the Wizard Knight is thrilling and helps to sustain an interest which rarely flags. E. A. Mason is responsible for the very good illustrations throughout the volume.

Froggy Folk. By G. E. H. (London: Grant Richards, 40 Leicester Square. 3s. 6d.)

Whether in respect to its coloured pictures or its clear letter-press, "Froggy Folk" is worthy of a permanent place in the series of "Dumpy Books" published by Mr. Grant Richards. The author's dual genius is so impartially divided that the fortunate girls and boys who receive the book, may be at a loss to decide whether the illustrations or the rhymes are more delightful. Both are exceedingly entertaining, not only on account of their genuine humour, but also because of their originality and quaintness. The interest in the doings of the froggy family can hardly even be stimulated by the knowledge that the King has been pleased to accept a copy of the work, and to thank the author for it.

Bo-Peep. (Cassell and Co., Limited.)

A capital children's gift book, full of pictures and short stories.

Just-So Stories. By Rudyard Kipling. (Macmillan and Co., Limited. 6s.)

This is a book in a class by itself, and many a grown-up reader will enjoy a hearty laugh on reading its entertaining pages. Various animals are dealt with, and to each is attached a clever illustration from the pen of the author. We are told how the leopard got his spots, and how the camel came by his hump, and what various other beasts did and did not do. There is a fair quantity of rhyme, and the best verses are those on page 195, in which every leading line of steamers plays a part. We can heartily recommend the stories to all our readers.

A Child at the Helm. By Winifred Graham. (George Newnes and Son. 3s. 6d.)

Adora's influence over others is the theme of this charming story. She is a life-like little character, and performs a distinct duty in her busy surroundings. Mr. H. M. Brock's illustrations leave nothing to be desired.

Two Little Travellers. By Ray Cunningham. (Thomas Nelson and Sons. 2s. 6d.)

This is quite one of the best children's books we have had under our notice this season. The adventures of the diminutive Darby and Joan, and their kidnapping by the rough circus owners, Joe and Moll, must have quickly dispelled any ideas they had of the Happy Land outside their aunt's house. It is a story that all little ones will thoroughly appreciate.

Peterkin. By Mrs. Molesworth. (Macmillan. 4s. 6d.)

This story is no exception to Mrs. Molesworth's invariably interesting books for the little ones. The tale turns on a parrot and the strange influence its power of speech has over Peterkin. The child's imagination, as regards the bird, helps to explain many curious coincidences. It is a book we heartily recommend to all who want a nice present for their young friends at Christmas time, and the illustrations by Mr. H. R. Millar materially enhance the merit of the story.

### BOOKS FOR GIRLS.

The New Pupil. By Raymond Jacberus. (Macmillan. 4s. 6d.)

An excellent story of a small girls' school, the new pupil being little Pollie Quebe, who had never before left her home in Italy. The story relates all the ups and downs of the little newcomer during her first term, and is told in a spirited and vivacious manner. We can recommend it to all girl readers who are still at school.

A Girl Capitalist. By Florence Bright. (Chatto and Windus. 6s.)

This is a bright and interesting novel which should find its way into many an appreciative girl reader's hands. The influence of the strike at the heroine's works upon her life of hitherto selfish enjoyment, and the consequent resignation of the manager, are well told. The plot, however, comes to rather a weak finish in Sarah's marriage with Richard Austen.

A Little Cockney. By S. G. (Thomas Nelson and Sons 1s. 6d.)

This is the history of a little girl's visit to her grandmother: the impressions made on her by all she sees and hears in the country are very prettily told.

### BOOKS FOR BOYS.

At the Point of the Sword. By H. Hayens. (Thomas Nelson and Sons. 5s.)

This book will be specially interesting to those who take an intelligent interest in the history of our own time. The scene is laid in the beginning of last century in Peru, and the story follows the career of a young Englishman who was born near Lima. The story deals with the successful overthrow by the patriot army of Spanish misrule, and some of the adventures are most exciting. The two incidents that strike the reader most forcibly are Juan's release by the Indians of the "Society of the Silver Key" from his imprisonment and his escape over the treacherous morass with the irrepressible Alzura. Parts of the story are tedious, and perhaps the plot is drawn out a little, but on the whole, combined with appropriate illustrations by R. P. R., we are bound to say the book would form a splendid Christmas present for any boy worthy of the name.

(*To be continued.*)

### Christmas Presents.

By our Shopping Correspondent.

The shops in London are full at present of tempting novelties, both useful and ornamental, to lure the Christmas shopper. But so much to choose from causes a bewilderment of ideas, and I am not sure that the stay-at-home shopper does not succeed best in the end. A few useful suggestions and a knowledge of the requirements of those on whom we mean to bestow our gifts is after all the best equipment for making a wise selection, for time is saved by this for both town and country dweller.

### AT MESSRS. PENBERTHY'S.

Messrs. Penberthy, of 390 Oxford Street, the well-known glovers, have most successfully laid themselves out this year to provide delightful gifts suitable to all manner of persons. Those who enter the shop to buy that useful and always acceptable present of gloves, must pause on their way to admire the charming feather fans, which are amongst Messrs. Penberthy's specialities. One of these is represented in our illustration, but of course this lacks the bright and soft tones of the feathers. These fans cost from half-a-crown upwards, and are of excellent taste and workmanship. Numerous other varieties of lovely fans are illustrated in Messrs. Penberthy's catalogue. I was much struck by some dainty handkerchiefs. At one corner a spray of tinted flowers is delicately outlined with embroidery. There are flowers of four shades

Serviceable Gloves.

Cholera Belt Combination.

Feather Fan.

to choose from, each with its spray of green leaves. These handkerchiefs are only 1s. $0\frac{1}{2}$d. each. There are numerous other designs in fancy handkerchiefs and suitable boxes to hold varying quantities. The Santoy bed jacket, lined and unlined, in soft stripes of silk and wool or all silk, and trimmed with silk, are most comfortable and sensible garments, which will appeal especially to the nurse off duty who likes her breakfast in bed. Amongst the more strictly necessary articles shown, are the Cholera belted combinations, hygienic, soft and well-fashioned; other warm undergarments of all kinds, and especially well-made nightgowns of nuns' veiling, so pretty that the idea of clumsiness usually associated with woollen garments is entirely banished. Hand-knitted gloves at 1s. 11d. are also good and useful.

### WATCHES AND JEWELLRY.

No doubt you have noticed an advertisement lately which has a quotation from Shakespeare: "Let's talk of Graves." Mr. J. G. Graves, of Sheffield, is able to supply a very large variety of watches and other jewellery at exceedingly low prices, and as they appear to be of good workmanship, and reliable as timekeepers, I should think nurses would be wise to send for particulars. There is one called the "Sister's" watch; it has a keyless movement, a white dial, and a strong engraved case; the price is 30s., or, a better quality, two guineas. There is also the "Nurse's" watch, warranted for five years; the dial is divided into minutes, seconds, and fourths of a second, for the greater accuracy of observation, and the price, in oxydised steel, is 25s., in sterling silver 30s. A cheaper watch still is one for 15s., recommended as sound. All the watches are examined and tested before they are sent out, and they are made in Mr. Graves' own workshops in Sheffield. Write to Messrs. J. G. Graves, Midland Direct Supply Warehouse, Sheffield, for a "Red Cross Department" catalogue.

### EAU DE COLOGNE.

Mühlens 4711 Eau de Cologne always is to my mind one of the nicest gifts that can be received. This season Messrs. Mühlens have introduced a handy little flat bottle with sprinkling top, which seems almost indispensable for travelling. The price is only 1s. The depôt is at 162 Bond Street, but it can be had almost everywhere where perfumes are sold. A visit to Bond Street reveals other delightful adjuncts of the toilette—fragrant perfumes such as Rhine violets, and all manner of enticing soaps and other things.

### CHOCOLATE FOR CHRISTMAS.

Messrs. Cadbury's chocolates are too well-known for excellence to need an advocacy on their merits, but we can suitably mention the form in which they have met the wants of Christmas purchasers. The "Tudor" chocolate box will appeal to all on account of its pretty taste. A long pure white box, is ornamented with the heraldic Tudor roses in red, white, green, and gold, and tied with green ribbons. The contents, of course, are delicious. To buy Messrs. Cadbury's chocolates is to encourage a home industry and secure wholesome and well-made sweetmeats.

### NOVELTIES IN NEEDLEWORK.

For dainty novelties in needlework, purchasers should wend their way to J. Harris and Sons, 25 Old Bond Street. There they will find an exhibition of beautiful stitchery applied to most practical and ornamental purposes. Photo frames, table centres, framed calendars, diaries, remembrancers, blotters, and a host of other articles are on exhibition, of original and artistic character. The delightful "Harris" linen is largely and effectively used in the work.

### TO REPLACE THE CHRISTMAS CARD.

The Christmas card is becoming more and more unpopular, yet it is not easy to replace it because it represents a souvenir at a small cost. The Mazawattee Company have solved the difficulty as far as children are concerned by introducing a penny Christmas gift, a chocolate encased in a neat little gold-coloured metal box containing a Christmas greeting. For twopence there is a similar box ornamented with a chromo-lithograph design upon the boxes The boxes would be an excellent idea for Christmas treats, when they would prove an interesting feature.

### A USEFUL AND PRETTY MATERIAL.

For an intending present of a warm blouse, useful bed jacket, or dressing gown, or for the dress of a little girl, I can confidently draw the attention of readers to Orlwoola. This is a fine, light, and soft material, somewhat after the style of delaine and nuns' veiling, but having a distinct characteristic of its own. It has been manufactured in such pretty designs, and the colours are so pleasing, that I recommend nurses to send to their drapers for patterns, if they cannot go personally to inspect it.

### EMPRESS SHORTBREAD.

A tin of shortbread is a convenient and suitable present at Christmas time, when all kinds of good fare receive a welcome and special attention. Any confectioner or grocer will order and send off tins of the excellent shortbread known as the "Empress," which is made by Messrs. Gray, Dunn and Company. The shortbread is done up in thin fluted cakes, about five inches long, and dusted with sugar.

### Appointments.

[No charge is made for announcements under this head, and we are always glad to receive, and publish, appointments. But it is essential that in all cases the school of training should be given.]

BIRMINGHAM INFIRMARY.—Miss Mabel Holberton has been appointed assistant matron, and Miss Adelaide Bottrill home sister. Miss Holberton was trained at the General Hospital, Cheltenham, where she also became sister of a male medical ward; superintendent nurse at the Derbyshire Asylum; ward sister at Birmingham Infirmary from March, 1898, to June, 1900; and home sister from June, 1900, to the present date. Miss Bottrill was trained at Birmingham Infirmary and has been sister of medical and surgical wards, and, for the last three years, theatre sister.

CANTERBURY WORKHOUSE INFIRMARY.—Miss Florence Delia Lewis has been appointed superintendent nurse. She was trained at Stockport Union Infirmary, and afterwards became staff nurse. She has also been charge nurse at the Metropolitan Asylums Board hospital ships, charge nurse of the Hertford and Ware Joint Hospital, and private nurse at Manchester and Surbiton.

CHESTERFIELD AND NORTH DERBYSHIRE HOSPITAL.—Miss Ada F. Maddock has been appointed sister. She was trained at the Infirmary, Leicester, and was afterwards sister at the Lewisham Infirmary.

HOSPITAL FOR SOLDIERS' WIVES AND CHILDREN, WOOLWICH.—Miss Rosalie M. Joyce has been appointed sister. She was trained at the General Infirmary, Gloucester, and holds the hospital certificates for maternity work.

MACCLESFIELD GENERAL INFIRMARY. — Miss Agnes Fletcher has been appointed sister. She was trained at the Royal Infirmary, Perth, where she was afterwards charge nurse. She has also been charge nurse at the City Hospital East, Liverpool.

PLAISTOW FEVER HOSPITAL, PLAISTOW.—Miss Jessie E. H. Ives has been appointed sister. She was trained at the Plaistow Fever Hospital and at the London Hospital, Whitechapel, E.

STAPLETON WORKHOUSE INFIRMARY, BRISTOL. — Miss Emma Marsh has been appointed charge nurse, and Miss Eva Gertrude Marks and Miss Charlotte A. Tuck assistant nurses. They were all trained at Stapleton Workhouse Infirmary, and all hold the L.O.S. certificate.

VICTORIA COTTAGE HOSPITAL, MARYPORT.—Miss Elizabeth Holliday has been appointed nurse-matron. She was trained at the General Infirmary, Bradford, and has since been night superintendent, assistant matron, and housekeeper [at the Royal Hospital for Women and Children, Bristol.

WARNEFORD HOSPITAL, LEAMINGTON. — Miss Marion Thomas has been appointed assistant matron. She was trained at the Warneford Hospital, Leamington, and afterwards became senior sister, also taking matron's duties during holiday time.

WEST CORNWALL MINERS' AND WOMEN'S HOSPITAL, REDRUTH.—Miss E. Williams and Miss F. Ibbottson have been appointed charge nurses. Miss Williams was trained at the Ashton-under-Lyne and District Infirmary, afterwards working as staff nurse and doing temporary sister's duties. Miss Ibbottson was trained at Sheffield Royal Infirmary, where she was staff nurse and took sister's holiday duty. Subsequently she was sister of the women's and children's wards, Blackburn Infirmary, charge nurse at the Metropolitan Asylums Board North-Western Hospital, Hampstead, and night charge at Hertford and Ware Joint Hospital. Miss Ibbottson holds the L O.S. certificate.

### Presentations.

MANCHESTER CHILDREN'S HOSPITAL.—On retiring from the matronship of the Manchester Children's Hospital, Pendlebury, Miss Turner received numerous valuable tokens of the affectionate regard and esteem to which she was held by those with whom she has worked so harmoniously for the past 15 years. The gifts were as follows:—The lay board, a beautiful opal brooch surrounded by 15 diamonds; a gold chain bracelet with heart attached, an umbrella, and a cheque. The medical board: a long gold chain. The resident medical officers: a silver-cased scent bottle. The secretary: a gold pencil case. The nursing staff: a diamond ring; and the household staff, a travelling clock. There were also many other small personal parting gifts. Miss Turner is leaving England on the 18th of this month for Australia.

### Crowned.

THERE blooms a lovely flower in Paradise,
In sweet profusive grace:
Sometimes across the gate a tendril lies
And as some radiant angel earthward flies
It clings about her face!

Then chancing by some sufferer's couch to stray
The angel stays her flight—
Pausing awhile, perhaps—as angels may
Unseen, unheard, to help a soul away
Up to a purer Light!

The pleading lips to perfect peace she kisses,
Then softly stooping down—
She takes the circling tendril from her tresses—
And on the pale and patient brow she presses—
A bright, immortal crown!

### "The Hospital" Convalescent Fund.

THE honorary secretary begs to acknowledge with thanks the receipt of 2s. 6d. from Nurse C. Jones, and of 2s. 6d. from Nurse Dudden, who is also thanked for her kind promise of a regular subscription, and thanks Sister Elizabeth for her donation of 10s.

### To Nurses.

WE invite contributions from any of our readers, and shall be glad to pay for "Notes on News from the Nursing World," or for articles describing nursing experiences, or dealing with any nursing question from an original point of view. The minimum payment for contributions is 5s., but we welcome interesting contributions of a column, or a page, in length. It may be added that notices of appointments, entertainments, presentations, and deaths are not paid for, but that we are always glad to receive them. All rejected manuscripts are returned in due course, and all payments for manuscripts used are made as early as possible after the beginning of each quarter.

### For Reading to the Sick.

"THE TESTIMONY OF THE LORD IS SURE."

"*O how sweet are Thy words.*"

Father of mercies, in Thy Word
What endless glory shines!
For ever be Thy Name adored
For these celestial lines.

Here springs of consolation rise
To cheer the fainting mind,
And thirsting souls receive supplies,
And sweet refreshment find.

Here the Redeemer's welcome Voice
Spreads heavenly peace around,
And life and everlasting joys
Attend the blissful sound.

Oh, may these heavenly pages be
My ever dear delight,
And still new beauties may I see,
And still increasing light.

Divine Instructor, gracious Lord,
Be Thou for ever near;
Teach me to love Thy sacred Word,
And view my Saviour here.

*Hymns Ancient and Modern, No.* 531.

The devotional study of the Holy Bible should be prefaced by prayer. "Open Thou mine eyes, that I may behold wondrous things out of Thy law." The best prayer to use by way of preparation is the Lord's Prayer, to which may be added the collect for the second Sunday in Advent.

The one object to be kept in view in our devotional reading is, that by patience and comfort of God's Holy Word, we may in all things be enabled to do His holy will, and ever hold fast the blessed hope of everlasting life, given to us in our Saviour Jesus Christ.

We read the history of the Old Testament and sing the Psalms, because they reveal to us the course by which God led His people like sheep, and the teaching which He gave to them age by age, until "the Word was made flesh, and dwelt among us."

There is not only a literal and historical, but also a mystical sense of Holy Scripture. In our devotional reading of the Old Testament, we should seek for this mystical sense. The mystical is not contrary to the literal sense, but derived from it, by those who have eyes to perceive it. If we believe that God caused all the Holy Scriptures to be written for our learning, and that they testify of Christ, it is reasonable to look for Christ everywhere in them.—*Vernon Staley.*

In order to understand and interpret the Old Testament aright, we must begin with the New Testament; and if we meet with difficulties in the Old Testament, let us consider them with reference to Christ, and if we see Him revealed in them, we have reason to think that we have found the solution of the difficulty. . . . The New Testament is enfolded in the Old, and the Old Testament is unfolded in the New.

*St. Augustine.*

### Notes and Queries.

**The Editor is always willing to answer in this column, without any fee, all reasonable questions, as soon as possible.**

**But the following rules must be carefully observed:—**

**1. Every communication must be accompanied by the name and address of the writer.**

**2. The question must always bear upon nursing, directly or indirectly.**

**If an answer is required by letter a fee of half-a-crown must be enclosed with the note containing the inquiry, and we cannot undertake to forward letters addressed to correspondents making inquiries. It is therefore requested that our readers will not enclose either a stamp or a stamped envelope.**

*Maternity.*

(88) A maternity nurse went to the house of her patient at the appointed time, and was there for three weeks. At the end of that time she was obliged to leave in order to attend another case for which she had been engaged previously. Can she claim full fees? *M. H.*

She can claim full fees for the three weeks she was in the house.

Could you tell me of any training school or teacher who would coach me for the certificate of the L. O. S. in Liverpool other than the Hospital for Women, Brownlow Hill?—*Anxious.*

Maternity nursing is taught in the Mill Road Infirmary, Liverpool, and at the Workhouse Infirmary, Brownlow Hill.

1. I should be glad to know if there is any place where I could get training for the L.O.S. free. 2. Also if there is an institute for English nurses in Florence or Rome.—*Nurse C.*

1. Some private homes give free training in maternity nursing in return for services. See advertisement columns. 2. Florence: Association of Trained Nurses and Masseuses, 7 Via Rondinelli. Rome: Anglo-American Nursing Home, 265 Via Nomentana.

*Hospital Training.*

(89) Can you tell me if the nurses who are trained in English hospitals receive as much, or more, medical instruction during their training as do the nurses trained in American hospitals? Can you tell me which is considered the best London hospital?—*Omega.*

The training in the best English hospitals is second to none.

Will you kindly tell me the name of some convalescent hospitals in London or the vicinity where I might apply for post as probationer?—*Rakii.*

Nurses are not trained in convalescent homes. See "The Nursing Profession: How and Where to Train" for particulars of training schools.

*Pamphlet.*

(90) I am anxious to hear of a small book or pamphlet which would give useful hints for a course of lectures on elementary nursing, to be delivered to a company of working women, and should be very glad if you could tell me what might be helpful.—*Nurse A.*

The only way to lecture effectually is to speak from one's own knowledge and experience. As a means of giving order and form to your lecture you would probably find Laurence Humphry's "Manual of Nursing" of considerable service.

*Scotland.*

(91) I am anxious to find employment in the beginning of next year in a bracing part of Scotland, either as nurse-companion or in a nursing home. Will you kindly give the address of a good Scotch paper in which to advertise, and advise me as to the best means of obtaining work?—*O. P. G.*

The *Scotsman* (London office, 45 Fleet Street, E.C.) is one of the most widely read of Scottish papers.

**Useful Handbooks for Nurses.**

"Nurses' Dictionary of Medical Terms." Cloth, 2s.; leather, 2s. 6d.; post free 2s. 8d.

"On Preparation for Operation in Private Houses." 6d.

"Hospital Sisters and their Duties." 2s. 6d.

"Medical Gymnastics, including the Schott (Nauheim) Movements." 2s. 6d.

"The Human Body." 5s.

"Practical Handbook of Midwifery." 6s.

"A Handbook for Nurses." (Illustrated.) 5s.

"Tendencies to Consumption: How to Counteract Them." 2s. 6d.

"Syllabus of Lectures to Nurses." 1s.

The above works are published by the Scientific Press, Ltd., and may be obtained through any bookseller or direct from the publisher, 28 and 29 Southampton Street, Strand, London, W.C.

## Figures and Tables

**Figure f1:**
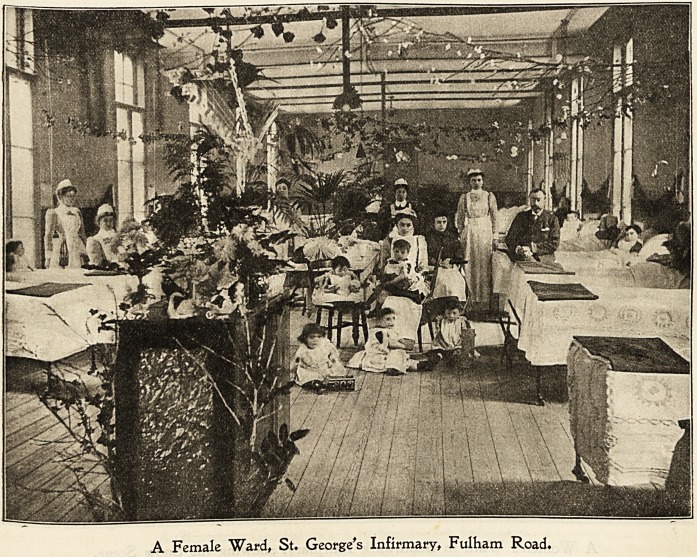


**Figure f2:**
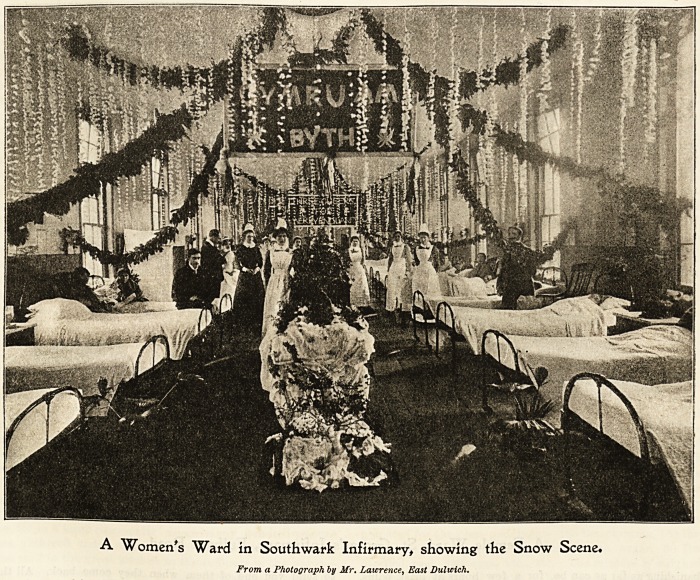


**Figure f3:**
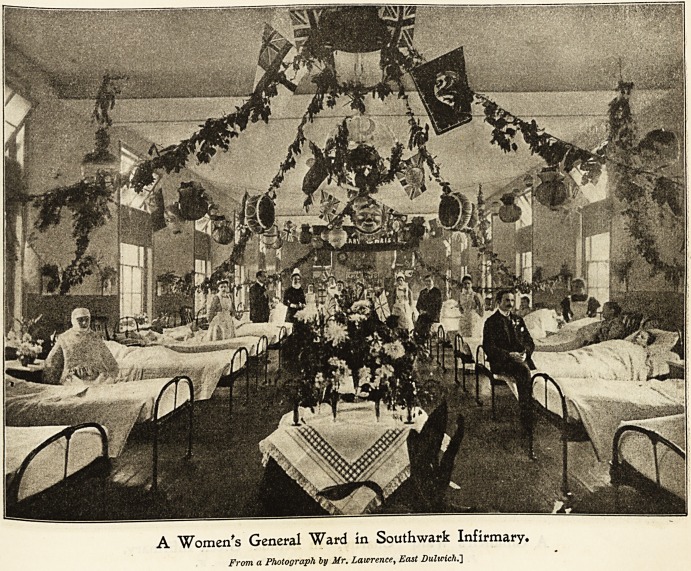


**Figure f4:**
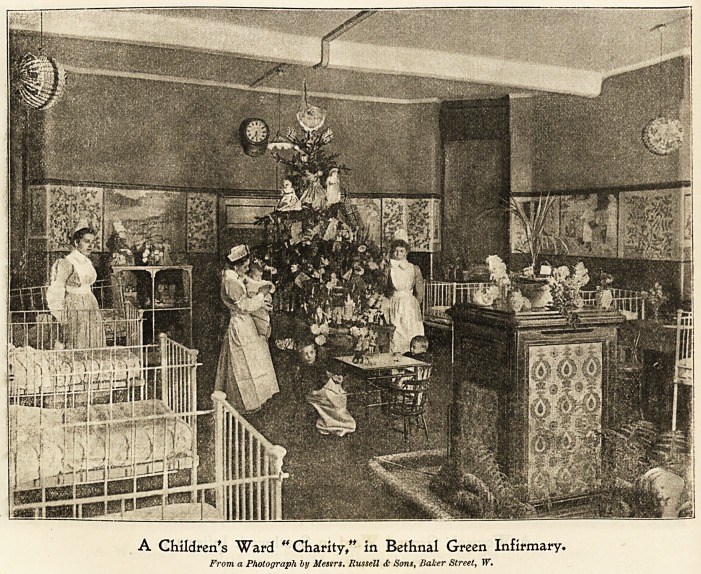


**Figure f5:**
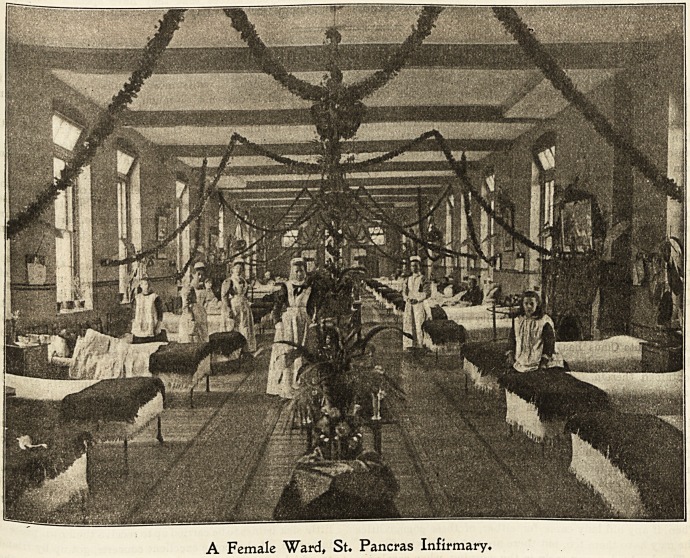


**Figure f6:**
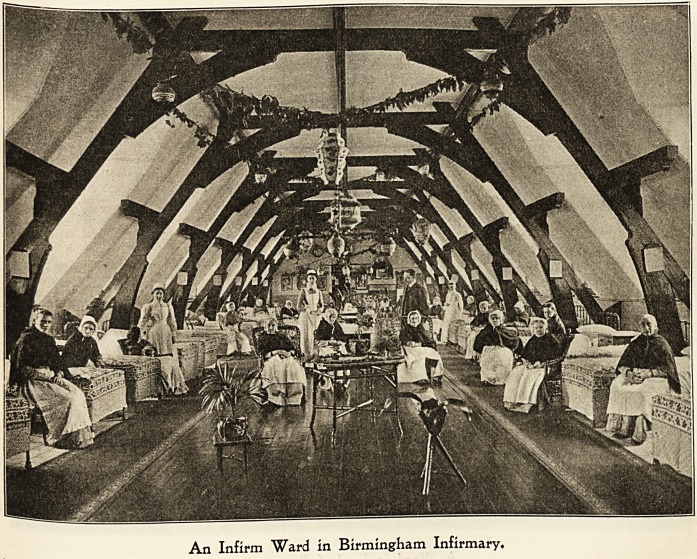


**Figure f7:**
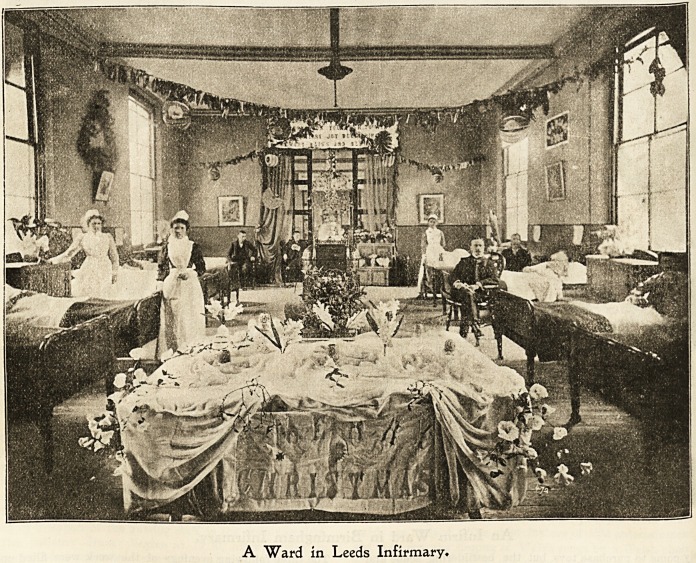


**Figure f8:**
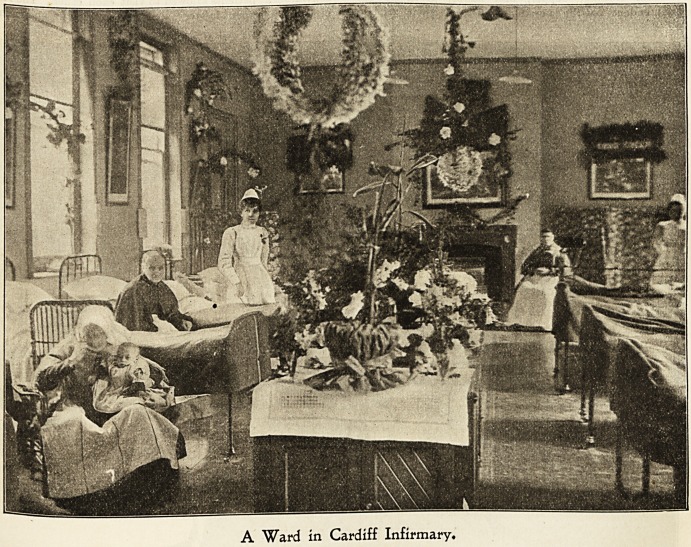


**Figure f9:**
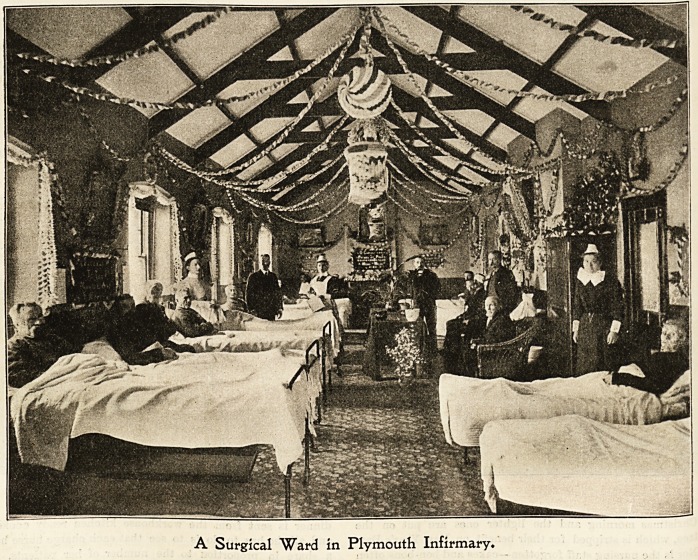


**Figure f10:**
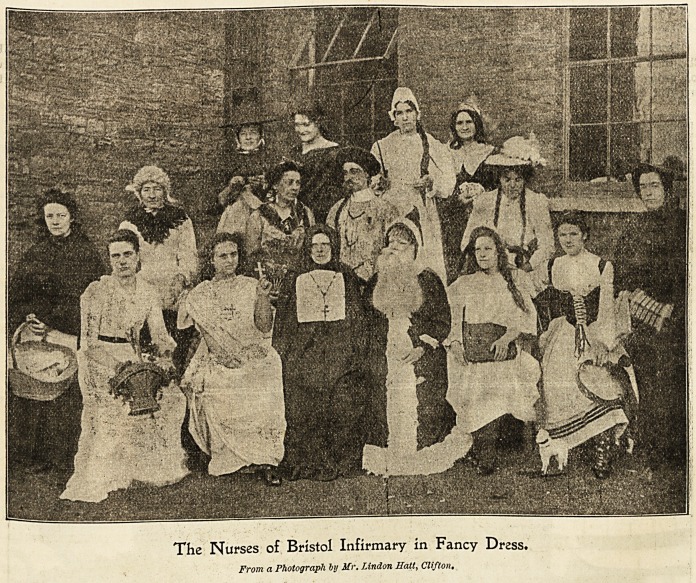


**Figure f11:**
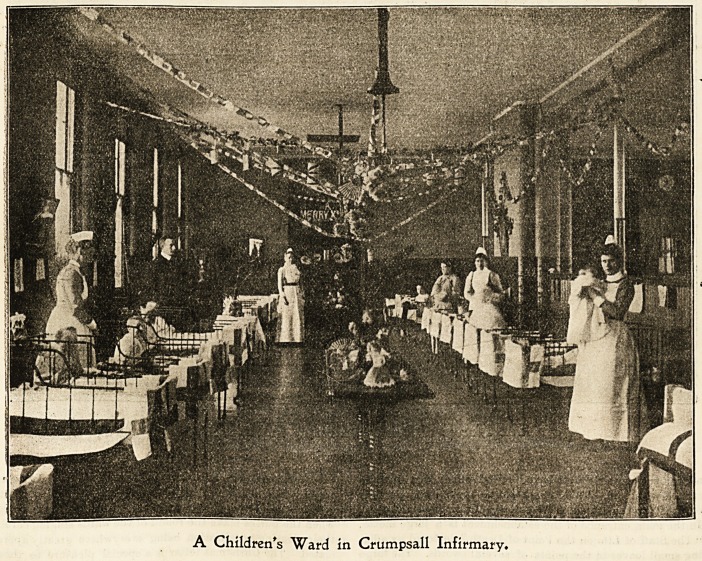


**Figure f12:**
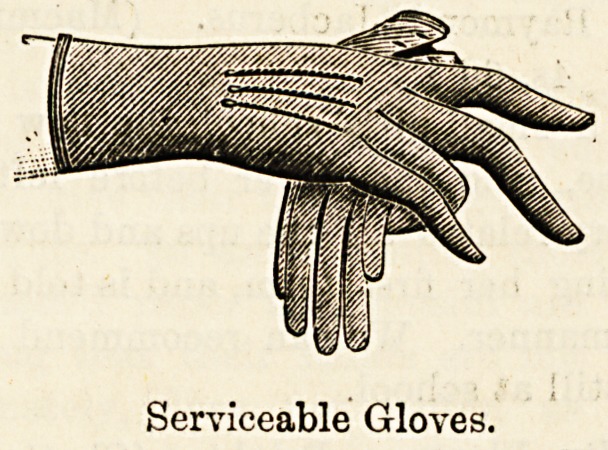


**Figure f13:**
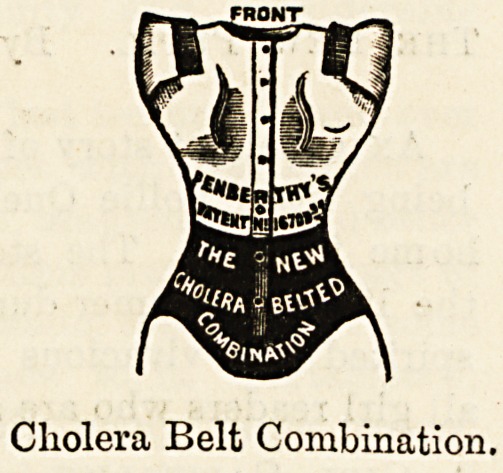


**Figure f14:**